# Association of Thyroid Diseases with Primary Extra-Thyroidal Malignancies in Women: Results of a Cross-Sectional Study of 6,386 Patients

**DOI:** 10.1371/journal.pone.0122958

**Published:** 2015-03-31

**Authors:** Natalie Prinzi, Salvatore Sorrenti, Enke Baldini, Corrado De Vito, Chiara Tuccilli, Antonio Catania, Carmela Coccaro, Marta Bianchini, Angela Nesca, Giorgio Grani, Renzo Mocini, Enrico De Antoni, Massimino D’Armiento, Salvatore Ulisse

**Affiliations:** 1 Department of Experimental Medicine, “Sapienza” University, Rome, Italy; 2 Department of Surgical Sciences, “Sapienza” University, Rome, Italy; 3 Department of Public Health and Infectious Diseases, “Sapienza” University, Rome, Italy; Uppsala University, SWEDEN

## Abstract

We here analyzed the prevalence of extra-thyroidal malignancies (EM) in 6,386 female patients affected by different thyroid disease (TD). At first, an age-matched analysis of EM in all patients was performed. We then evaluated EM prevalence in four TD diagnostic categories: non-nodular TD (n = 2,159); solitary nodule (n = 905); multinodular TD (n = 2,871); differentiated thyroid cancers (n = 451). Finally, patients were grouped based on the absence (n = 3,820) or presence of anti-thyroglobulin (TgAb) and/or anti-thyroperoxidase (TPOAb) (n = 2,369), or anti-Thyroid Stmulating Hormone (TSH) receptor autoantibodies (n = 197). A total of 673 EM were recorded. EM prevalence in TD patients was higher compared to the general population (Odds Ratio, OR 3.21) and the most frequent EM was breast cancer (OR 3.94), followed by colorectal (OR 2.18), melanoma (OR 6.71), hematological (OR 8.57), uterus (OR 2.52), kidney (OR 3.40) and ovary (OR 2.62) neoplasms. Age-matched analysis demonstrated that the risk of EM was maximal at age 0–44 yr (OR 11.28), remaining lower, but significantly higher that in the general population, in the 45–59 and 60–74 year age range. Breast and hematological malignancies showed an increased OR in all TD, while other cancers associated with specific TD. An increased OR for melanoma, breast and hematological malignancies was observed in both TPOAb and/or TgAb autoantibody negative and positive patients, while colorectal, uterus, kidney and ovary cancers showed an increased OR only in thyroid autoantibody negative patients. In conclusions, women affected by both benign and malignant TD, especially at a younger age and in absence of thyroid autoimmunity, have an increased risk of developing primary EM, thus requiring a careful follow-up and surveillance.

## Introduction

Thyroid diseases are more frequent in females than in males [1]. Iodine deficiency is the world’s most common cause of thyroid disease leading to hypothyroidism and diffuse or nodular goiter. In iodine-repleted areas thyroid autoimmunity, causing either chronic lymphocytic thyroiditis or Graves’ disease, represents the main type of thyroid disease [1]. The prevalence of nodular thyroid disease varies according to the diagnostic methods employed and the populations analyzed, being higher in areas with low iodine intake [1–4]. Although the majority of thyroid nodules are benign tumors, about 5% of them harbors a malignant lesion derived from the transformation of parafollicular cells or thyrocytes which generate medullary thyroid cancer (MTC) and well-differentiated thyroid cancer (DTC), respectively. The latter comprises the papillary (PTC), which account for about 90% of all thyroid carcinomas, and follicular (FTC) histotypes [5–6]. Despite the relevant progress made in the comprehension of the molecular pathogenesis of both benign and malignant thyroid tumors, much more needs to be learned regarding their etiology [7–9]. To this regard, accumulated data drawn from large-scale case studies documenting a 30% increase in the risk of a second primary thyroid cancer in patients who have had other primary malignancies are of interest [10–13]. Correspondingly, a 20–42% increased risk of second primary malignancies in patients affected by DTC has been reported [14–21]. In particular, for some cancers (e.g. prostate, kidney and adrenal gland) the risk was statistically higher within a year following the diagnosis of DTC, while for other cancers (e.g. colon, rectum and breast) the risk increased with the duration of the follow-up [22]. Whether the effects of treatments, environmental or genetic factors are responsible for the association between DTC and other cancers, is still a matter of debate [10–13]. As to the prevalence of EM in patients affected by benign thyroid disease, few and conflicting data have been reported, mainly regarding breast cancer [23–27]. Herein, on the basis of a cross-sectional study of 6,386 female patients, we evaluated the association of benign and malignant thyroid disease with other primary EM, compared to the general population of the same geographical area.

## Patients and Methods

### Case study

In this cross-sectional study we included 6,386 consecutive female patients (mean age 51.2 yr, age range 18–92 yr) affected by various thyroid disease diagnosed according to standard criteria [28–30] undergoing their first observation at the Thyroid Unit of the Umberto I Hospital of Rome, Italy, between 2000 and 2011. All the patients came from central-southern Italy, an area characterized by a moderate iodine deficiency [31]. Patients gave the written informed consent, and their records were de-identified prior to the analysis. The ethics committee of the Umberto I Hospital of Rome approved the study (n°. 2615/17.01.2013). Patients with MTC and those with a less than one year follow-up were excluded from the case study. For each patient, age, anti-thyroglobulin (TgAb), anti-thyroperoxidase (TPOAb), and anti-TSH receptor (TSHRAb) autoantibodies, and the presence of one or more primary EM were recorded. Prevalence of EM in the general population of the central-southern Italy was obtained from the relative regional cancer registries [32]. At first, an age-matched analysis of EM in all the thyroid disease patients concerned was performed (Table 1). Then, we evaluated EM prevalence in four thyroid disease diagnostic categories, which included: 1) 2,159 patients with non-nodular thyroid disease (NNTD) comprising chronic lymphocytic thyroiditis, non-autoimmune hypothyroidisms, and Graves’ diseases; 2) 905 patients with solitary thyroid nodule (SN); 3) 2,871 patients with multinodular thyroid disease (MNTD); 4) 451 patients affected by differentiated thyroid cancers (DTC) (Table 2). Finally, the patients were divided into 3 groups based on the absence (n = 3,820) or presence of anti-thyroglobulin (TgAb) and/or anti-thyroperoxidase (TPOAb) (n = 2,369), or anti-TSH receptor (TSHRAb) autoantibodies (n = 197) (Table 3). Sixty-five low frequency EM (with no more than 10 cases each) were grouped together and indicated as other EM. These include cancer of: bladder (n = 10), lung (n = 9), cervix (n = 8), pancreas (n = 8), stomach (n = 7), central nervous system (n = 5), liver (n = 5), larynx (n = 2), bone (n = 2), sarcoma (n = 2), external genitals (n = 2), salivary gland (n = 1), gallbladder (n = 1), hepatopancreatic ampulla (n = 1), mesothelioma (n = 1) and ameloblastic cancer (n = 1). The hematological malignancies included leukemia (n = 17), Hodgkin lymphoma (n = 13) and non-Hodgkin lymphoma (n = 20).

**Table 1:** Age and Extra-Thyroidal Malignancies in the 6,386 Female Patients Included in the Study.

***Age range***	***0–44***	***45–59***	***60–74***	***>75***	***All***
***N*. *of TD patients***	2,168	2,169	1,169	380	6,386
***Extra-thyroidal malignancies***					
**Breast**					
*General population prevalence*	149	1,940	3,557	3,988	1,473
*EM patient*: *expected/observed*	3.2/59	42.1/172	41.6/106	15.2/18	94.1/355
OR (95% CI)	18.747(13.581–25.595)	4.354(3.689–5.135)	2.704(2.188–3.325)	1.197(0.701–1.922)	3.937(3.490–4.441)
*P* values	**<0.0001**	**<0.0001**	**<0.0001**	0.4567	**<0.0001**
**Colorectal**					
*General population prevalence*	17	270	1,015	1,964	447
*EM patient*: *expected/observed*	0.4/3	5.8/23	11.9/28	7.5/8	28.5/62
OR (95% CI)	8.150(1.529–28.205)	3.959(2.462–6.081)	2.393(1.575–3.499)	1.073(0.459–2.141)	2.183(1.644–2.857)
*P* values	**0.0083**	**<0.0001**	**<0.0001**	0.8430	**<0.0001**
**Melanoma**					
*General population prevalence*	52	169	231	255	127
*EM patient*: *expected/observed*	1.1/15	3.7/18	2.7/15	0.9/6	8.1/54
OR (95% CI)	13.391(6.990–24.196)	4.943(2.856–8.071)	5.614(3.082–9.492)	6.275(2.267–13.973)	6.707(4.779–9.303)
*P* values	**<0.0001**	**<0.0001**	**<0.0001**	**<0.0001**	**<0.0001**
**Hematological**					
*General population prevalence*	47	106	164	182	92
*EM patient expected/observed*	1.0/24	2.3/9	1.9/15	0.7/2	5.9/50
OR (95% CI)	23.806(13.895–39.820)	3.927(1.745–7.752)	7.913(4.316–13.490)	2.902(0.348–10.704)	8.570(5.942–12.236)
*P* values	**<0.0001**	**<0.0001**	**<0.0001**	0.1543	**<0.0001**
**Uterus**					
*General population prevalence*	16	227	771	1,018	287
*EM patient*: *expected/observed*	0.3/13	4.9/16	9.0/13	3.9/4	18.3/46
OR (95% CI)	37.697(16.659–83.680)	3.266(1.832–5.433)	1.447(0.765–2.500)	1.034(0.280–2.679)	2.521(1.803–3.455)
*P* values	**<0.0001**	**<0.0001**	0.1861	0.7976	**<0.0001**
**Kidney**					
*General population prevalence*	20	81	216	310	97
*EM patient*: *expected/observed*	0.4/3	1.7/9	2.5/7	1.2/2	6.2/21
OR (95% CI)	6.927(1.317–23.382)	5.140(2.266–10.263)	2.783(1.103–5.850)	1.701(0.204–6.241)	3.398(2.012–5.493)
*P* values	**0.0123**	**<0.0001**	**0.0055**	0.3308	**<0.0001**
**Ovary**					
*General population prevalence*	29	168	285	236	120
*EM patient*: *expected/observed*	0.6/4	3.6/7	3.3/6	0.9/3	7.7/20
OR (95% CI)	6.372(1.626–18.168)	1.924 (0.761–4.058)	1.805(0.656–3.989)	3.364(0.686–10.024)	2.615(1.542–4.224)
*P* values	**0.0051**	0.0847	0.1474	0.0631	**<0.0001**
***Others EM***					
*General population prevalence*	14	49	101	168	48
*EM patient*: *expected/observed*	0.3/12	1.1/24	1.2/25	0.6/4	3.1/65
OR (95% CI)	39.571(16.775–92.681)	22.823(13.366–38.013)	21.615(13.309–33.905)	6.322(1.694–16.616)	21.413(14.506–31.780)
*P* values	**<0.0001**	**<0.0001**	**<0.0001**	**<0.0001**	**<0.0001**
**All**					
*General population prevalence*	576	3,893	8,111	10,602	3,544
*EM patient*: *expected/observed*	12.5/133	84.4/278	94.8/215	40.3/47	226.3/673
OR (95% CI)	11.281(9.225–13.750)	3.629(3.180–4.141)	2.553(2.192–2.973)	1.190(0.857–1.620)	3.206(2.937–3.500)
*P* values	**<0.0001**	**<0.0001**	**<0.0001**	0.2644	**<0.0001**

The total number of thyroid disease patients with extra-thyroidal cancer was 629, of whom 38 patients had two extra-thyroidal primary cancers and 3 patients had three extra-thyroidal cancers for a total of 673 malignancies. The general population prevalence values are referred per 100,000 persons, from which the expected cases in the different patient’s groups were estimated.

**Table 2:** Thyroid Diseases, Age and Extra-Thyroidal Malignancies in the 6,386 Female Patients Included in the Study.

	***NNTD***	***SN***	***MNTD***	***DTC***	***All***
***N*. *of patients***	2,159	905	2,871	451	6,386
***Mean age (yr)±SD***	47±15.4	50±14.7	55±13.9	48.2±14.5	51.2±15
***Age range***	18–91	18–85	18–89	18–92	18–92
***Extra-thyroidal malignancies*** *(prevalence in the general population/100*,*000)*					
**Breast** (1473)					
expected/observed	31.8/73	13.3/48	42.3/209	6.6/25	94.1/355
OR (95% CI)	2.341(1.817–2.975)	3.746(2.728–5.038)	5.221(4.507–6.117)	3.925(2.504–5.898)	3.937(3.490–4.441)
*P* values	**<0.0001**	**<0.0001**	**<0.0001**	**<0.0001**	**<0.0001**
**Colorectal** (447)					
expected/observed	9.7/17	4/4	12.8/38	2.0/3	28.5/62
OR (95% CI)	1.760(1.014–2.857)	0.984(0.266–2.547)	2.974(2.073–4.156)	1.485(0.304–4.395)	2.183(1.644–2.857)
*P* values	**0.0210**	0.9749	**<0.0001**	0.4937	**<0.0001**
**Melanoma** (127)					
expected/observed	2.7/13	1.1/6	3.6/33	0.6/2	8.1/54
OR (95% CI)	4.764(2.465–8.459)	5.249(1.886–11.784)	9.144(6.026–13.528)	3.503(0.418–12.983)	6.707(4.779–9.303)
*P* values	**<0.0001**	**<0.0001**	**<0.0001**	0.1147	**<0.0001**
**Hematological** (92)					
expected/observed	2/16	0.8/6	2.6/23	0.4/5	5.9/50
OR (95% CI)	8.108(4.441–13.906)	7.248(2.586–16.441)	8.770(5.290–13.995)	12.174(3.843–29.645)	8.570(5.942–12.236)
*P* values	**<0.0001**	**<0.0001**	**<0.0001**	**<0.0001**	**<0.0001**
**Uterus** (287)					
expected/observed	6.2/10	2.6/7	8.2/24	1.3/5	18.3/46
OR (95% CI)	1.617(0.766–3.022)	2.708(1.076–5.675)	2.923(1.843–4.555)	3.895(1.249–9.261)	2.521(1.803–3.455)
*P* values	0.1325	**0.0069**	**<0.0001**	**0.0012**	**<0.0001**
**Kidney** (97)					
expected/observed	2.1/3	0.9/2	2.8/12	0.4/4	6.2/21
OR (95% CI)	1.433(0.290–4.318)	2.281(0.272–8.483)	4.328(2.157–7.911)	9.216(2.451–24.514)	3.398(2.012–5.493)
*P* values	0.5374	0.2356	**<0.0001**	**<0.0001**	**<0.0001**
**Ovary** (120)					
expected/observed	2.6/6	1.1/0	3.4/11	0.5/3	7.7/20
OR (95% CI)	2.320(0.834–5.205)	n.d.	3.201(1.555–5.944)	5.574(1.129–16.779)	2.615(1.542–4.224)
*P* values	**0.0386**	n.d.	**0.0001**	**0.0010**	**<0.0001**
**Others EM** (48)					
expected/observed	1/18	0.4/8	1.4/36	0.2/3	3.1/65
OR (95% CI)	17.507(9.568–30.710)	18.571(7.564–39.736)	26.442(16.643–41.662)	13.944(2.768–43.515)	21.413(14.506–31.780)
*P* values	**<0.0001**	**<0.0001**	**<0.0001**	**<0.0001**	**<0.0001**
**All** (3544)					
expected/observed	76.5/156	32.0/81	101.7/386	16.0/50	226.3/673
OR (95% CI)	2.120(1.788–2.511)	2.675(2.097–3.174)	4.228(3.772–4.738)	3.394(2.472–4.571)	3.206(2.937–3.500)
*P* values	**<0.0001**	**<0.0001**	**<0.0001**	**<0.0001**	**<0.0001**

NNTD, non-nodular thyroid disease; SN, solitary nodule; MNTD, multinodular thyroid disease; DTC, differentiated thyroid cancer; n.d., not determinable; 95% CI, 95% confidence interval. The expected cases in the different patient groups were estimated according to the prevalence of the different tumors occurring in the general population.

**Table 3:** Prevalence of Extra-Thyroidal Malignancies in Patients Positive (n = 2,369) or Negative (n = 3,820) for TgAb and/or TPOAb.

***Extra-thyroidal malignancies***	***Number of EM in TgAb and/or TPOAb positive***	***Odds ratio (95% CI)***	***P value***	***Number of EM in TgAb and TPOAb negative***	***Odds ratio (95% CI)***	***P value***
**Breast**	102	3.010 (2.428–3.711)	**<0.0001**	246	6.604 (3.996–5.303)	**<0.0001**
**Colorectal**	15	1.419 (0.786–2.371)	0.1815	45	2.655 (1.906–3.619)	**<0.0001**
**Melanoma**	19	6.358 (3.700–10.364)	**<0.0001**	35	7.272 (4.846–10.661)	**<0.0001**
**Hematological**	21	9.713 (5.731–15.764)	**<0.0001**	28	8.012 (5.049–12.374)	**<0.0001**
**Uterus**	9	1.325 (0.600–2.555)	0.4052	36	3.305 (2.266–4.693)	**<0.0001**
**Kidney**	2	0.870 (0.104–3.231)	0.5975	18	4.873 (2.772–8.128)	**<0.0001**
**Ovary**	6	2.113 (0.760–4.792)	0.0732	13	2.842 (1.469–5.053)	**0.0002**
**Others EM**	20	17.729 (9.951–30.497)	**<0.0001**	45	24.822 (16.136–38.119)	**<0.0001**
**All**	194	2.448 (2.099–2.854)	**<0.0001**	466	3.781 (3.408–4.195)	**<0.0001**

Statistical analysis was performed on all patients excluding those with TSHRAb. 95% CI, 95% confidence interval.

### Statistical analysis

The prevalence of each EM was determined for the patients taken as a whole or divided into different sub-groups. The Chi square test, the Fisher exact test and the prevalence odds ratio (OR) along with the 95% confidence interval (95% CI) were calculated to assess the association between all categories of thyroid disease patients and EM using STATA, version 12 (College Station, Texas, Stata Corporation). The prevalence odds ratio was used since, in this cross-sectional study, it represents the best measure of the association between thyroid disease and extra-thyroidal malignancies. The results were considered statistically significant when the p value was <0.05.

## Results

### Prevalence of EM in thyroid disease patients

The prevalence of EM in the general population of central-southern Italy is 3,544/100,000 (3.5%) [32]. As described in Table 1, we analyzed 6,386 consecutive female patients affected by various thyroid disease. Of the total, 629 (9.8%) patients showed EM, 38 of whom had two and 3 had three EM, which brought the total EM count to 673. Of these, 489 had been diagnosed before (range 2–41 yr, median 5 yr), 97 simultaneously (within 1 year before or after), and 87 after (2–36 yr, median 4 yr) being diagnosed with thyroid disease. The most frequently encountered EM was breast cancer with 355 cases (52.75% of all EM), followed by colorectal cancer with 62 cases (9.21%), melanoma with 54 cases (8.02%), hematological malignancies with 50 cases (7.43%), cancer of the uterus with 46 cases (6.84%), kidney cancer with 21 cases (3.12%) and ovary cancer with 20 cases (2.97%). The overall prevalence of EM in thyroid disease patients is significantly (OR 3.21, p<0.0001) greater than that recorded for the general population (see last column of Table 1).

### Age-matched association of thyroid disease and EM

**Fig 1:**
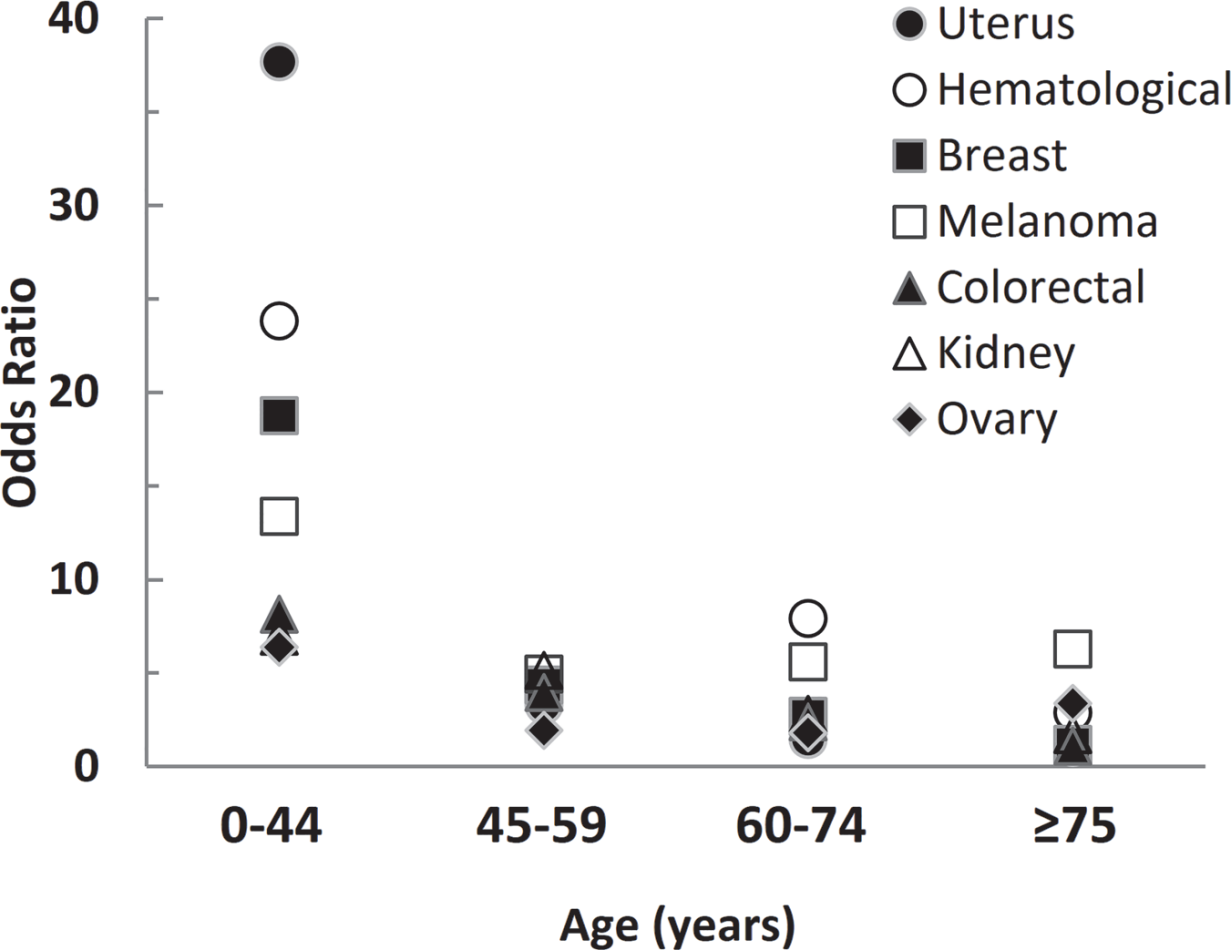
Odd ratio of various primary extra-thyroidal malignancies at different ages in 6,389 female patients affected by benign or malignant thyroid diseases.

The age-matched analysis demonstrated that the risk of EM was maximal for the 0–44 yr age range, with an OR of 11.28 (p<0.0001), spanning from 6.37 for ovary cancer to 37.70 for cancer of the uterus (Table 1 and Fig 1). The OR for all EM remains significantly (p<0.0001) higher for the 45–59 yr and 60–74 yr age ranges, being 3.63 and 2.55, respectively. In patients older than 75, with the exception of melanoma, the OR for EM (OR 1.19) was not significantly different from that of the general population (Table 1).

### Association of specific thyroid disease with EM

When patients affected by thyroid disease were divided into 4 diagnostic groups, it was found that only breast cancer and hematological malignancies had a significantly increased OR in all categories of thyroid disease patients (Table 2). As regards other cancer types, melanoma associated with NNTD, SN and MNTD; colorectal cancer with NNTD and MNTD; ovary cancer with NNTD, MNTD and DTC; cancer of the uterus with SN, MNTD and DTC and kidney cancer with MNTD and DTC (Table 2).

### Thyroid autoantibodies and EM

Of the 6,386 patients, 2,369 (37.09%) were positive for TgAb, and/or TPOAb, and 197 (3.08%) for TSHRAb, while the remaining 3,820 patients were negative. The 197 patients with TSHRAb showed an increased risk of EM (OR 1.923, CI 1.004–3.375, p = 0.0206), compared to the general population. In particular, among the patients affected by Graves’ disease 13 EM were recorded including neoplasms for breast (n = 7), colon (n = 2), kidney (n = 1), uterus (n = 1), ovary (n = 1) and hematological malignancy (n = 1). Given the low number of patients and the paucity of the EM encountered, no further analyses were performed on this group. Patients with TgAb and/or TPOAb revealed an increased risk for melanoma, breast cancer and hematological malignancies (Table 3). On the other hand, patients negative for thyroid autoantibodies presented an increased risk of all types of EM (Table 3). Finally, the OR for breast, colorectal, uterus and kidney cancers were significantly lower in thyroid autoantibody positive patients, with respect to the negative ones (Table 4).

**Table 4:** Differences in the Prevalence of Extra-Thyroidal Malignancies in Patients Positive (n = 2,369) or Negative (n = 3,820) for TgAb and/or TPOAb.

	**Number of EM**			
***Extra-thyroidal malignancies***	**TgAb and/or TPOAb positive**	**TgAb and TPOAb negative**	***Odds ratio (95% CI)***	***P value***
**Breast**	102	246	0.654 (0.511–0.832)	**0.0004**
**Colorectal**	15	45	0.535 (0.276–0.980)	**0.0335**
**Melanoma**	19	35	0.874 (0.471–1.576)	0.6387
**Hematological**	21	28	1.211 (0.652–2.516)	0.5079
**Uterus**	9	36	0.401 (0.170–0.850)	**0.0114**
**Kidney**	2	18	0.178 (0.020–0.745)	**0.0062**
**Ovary**	6	13	0.744 (0.231–2.100)	0.3642
**Others EM**	20	45	0.714 (0.398–1.238)	0.2106
**All**	194	466	0.642 (0.536–0.768)	**<0.0001**

Statistical analysis has been performed on all the patients excluding those with TSHRAb. 95% CI, 95% confidence interval.

## Discussion

Epidemiological studies aimed at defining the association of thyroid disease with extra-thyroidal malignancies (EM) have led to considerable interest in the possibility of revealing common genetic and environmental factors underlying disease aetiology and progression [10–23]. In particular, a number of different studies have highlighted the association between thyroid cancers and other primary EM, including cancers of the oral cavity, pharynx, salivary gland, stomach, colorectum, breast, ovary, uterus, kidney, brain, adrenal gland, non-Hodgkin lymphoma, and leukaemia, occurring either before or after diagnosis of thyroid cancer [10–22]. Regarding the risk of EM in benign thyroid disease, few and conflicting results have been reported, mainly regarding breast cancer [23–25]. This prompted us to analyze the relationship of EM not only with malignant thyroid disease, but also with benign thyroid disease. Furthermore, these associations were evaluated independently of the timing of thyroid disease diagnosis, because most thyroid disease, including carcinomas, are characterized by a slow progression that may take years to become clinically manifest and, hence, diagnosed. Moreover, this agrees with the Ronckers and colleagues [20] report demonstrating that the association between thyroid cancer and EM exists regardless of which cancer occurred first. Our results demonstrated that women affected by thyroid disease, considered as a whole, have an increased risk of EM (OR 3.21) compared to the general female population. Breast cancer was the most frequent EM observed, and the highest OR was found for hematological malignancies (OR 8.5), followed by melanoma (OR 6.7) and breast (OR 3.9) cancers. Age-matched analysis demonstrated that the highest OR (11.3) for EM occurred at an early age (0–44 yr), to decline at an older age. By dividing patients into four diagnostic categories (i.e. NNTD, SN, MNTD, and DTC), we observed that patients affected by non-malignant thyroid disease have an increased risk for EM. In particular, while melanoma and colorectal cancer associate with benign thyroid disease only, breast cancer and hematological malignancies associated with both benign and malignant thyroid disease. As regards DTC, our data confirm previous observations showing a significant relationship between DTC and hematological malignancies, kidney, ovary, uterus and breast cancers [10–22].

It has been suggested that the long-term carcinogenic effects of specific cancer treatments might be responsible for a second primary cancer. To this regard, several studies evaluating I^131^ treatment in thyroid cancer patients as a possible cause of increased risk of second primary EM have produced conflicting results [14–22]. In particular, in some studies a 30–42% increased risk of second primary malignancy attributed to I^131^ treatment has been reported, while in different studies no correlation between the exposure to I^131^ treatment and second primary malignancies could be appreciated [16–18, 20, 21]. Whether anticancer treatments of EM, in particular external beam radiation, may cause second primary thyroid cancers is also a matter of debate (11–13, 20]. The observations reported here regarding the association of EM not only with thyroid cancer but also with benign thyroid disease seem to suggest that factors other than oncologic treatment may play a role in the initiation and progression of second primary malignancies. In this context, the association of benign thyroid disease with breast cancer has been extensively investigated, although the findings have proven controversial [23–25]. An earlier meta-analysis by Sarlis and colleagues [24] found no association between autoimmune *thyroid disease* and breast cancer. More recently, 28 studies were reviewed in a meta-analysis by Hardefeldt and colleagues [23] showing an increased risk of breast cancer in patients with autoimmune thyroid disease. We recently showed that the OR for breast cancer increased in both thyroid autoantibody positive and negative patients [25]. However, the OR was significantly lower in thyroid autoantibody positive patients, compared to negative ones [25]. These results are confirmed in the present study performed on larger case series. In addition, we showed here that while thyroid autoantibody negative patients had an increased OR for all EM analyzed, in TgAb and/or TPOAb positive patients a significant increase in the OR was found only for breast cancer, melanoma and hematological malignancies. This is in agreement with previous findings showing that the development of thyroid autoimmunity in cancer patients receiving immunotherapy is associated with better outcome [33]. Taken on the whole, these observations indicate a protective role of thyroid autoantibodies *versus* EM, and support the clinical evidence that breast cancer patients positive for TPOAb have better disease-free interval and overall survival [23–25, 34]. Finally, in agreement with previous studies, we demonstrated an increased risk of EM in TSHRAb positive patients [26, 35].

The molecular links between thyroid disease and breast cancer remain unidentified, and different explanations have been proposed, such as the promoter role of sodium/iodide symporter, as expressed in both breast and thyroid tissues, or the presence of progesterone and estrogen receptors identified in the cytosol of tumor thyroid tissue, but not in normal tissue [36–42]. In addition, it has been documented that: i) the expression of thyroid hormone (TH) receptors is deregulated in primary and metastatic breast cancer cells; ii) TH may bind and activate the estrogen receptor in breast cancer cells; iii) TH level positively correlates with breast cancer risk; iv) TH affect estrogen production as well as estrogen receptor levels [36–42]. Based on this evidence, it may be speculated that at an earlier age, where the association between thyroid disease and breast cancer is highest (OR 18.8), estrogens and TH may act in concert to promote breast cancer progression. On the contrary, in older women, low-levels of free T4 represent an independent risk factor for breast cancer and this was confirmed by the finding that levothyroxine treatment improves overall survival [43, 44].

In conclusion, we demonstrated that women affected by both benign and malignant thyroid diseases, especially at a younger age and in absence of thyroid autoimmunity, have an increased risk of developing primary extra-thyroidal malignancies, thus requiring a very careful follow-up and surveillance. These observations should warrant the creation of regional and/or national registries to confirm these findings and to facilitate the identification of common genetic and environmental factors underlying such disease associations.
